# Effect of nano-encapsulated (-) epigallocatechin gallate on triglyceride accumulation in 3T3-L1 adipocytes

**DOI:** 10.1186/1753-6561-6-S3-P55

**Published:** 2012-06-01

**Authors:** Priyanka Bapat, Shu Wang

**Affiliations:** 1 Nutrition, Hospitality & Retailing Department, Texas Tech University, Lubbock, Texas, 79409, USA

## Background

Obesity is considered the major risk factor for type 2 diabetes mellitus (T2DM), cardio-vascular diseases like hypertension and atherosclerosis [1]. Catechins are the major source of flavonols and they are comprised of various types of catechins such as epigallo catechin 3 gallate (EGCG), epigallocatechin (EGC), epicatechin gallate (ECG) [2]. Numbers of *in vivo* studies have reported weight loss after drinking green tea for several months. To support these *in vivo* findings, many *in vitro* studies demonstrated effect of pure EGCG which is an important biologically active green tea catechin in reducing triglyceride accumulation in adipocytes [3]. However, these anti-adipogenic properties of EGCG are greatly dependent on bio-availability which is very low in animals as well as in humans. One study showed that, peak plasma concentrations in humans were 1.3 µM for EGCG after a single dose of 1.5 mmol through oral administration [4]. Nano-encapsulated EGCG (Nano-EGCG) may help to increase bioavailability and to exert maximum anti-adipogenic effects.

## Materials and methods

We synthesized Nano-EGCG using sonication method. Pure EGCG (95%) was bought from SIGMA Company. Nano-EGCG was dissolved in phosphate buffered saline (1XPBS) and was stored at 4 degree Celsius. 3T3-L1 Preadipocytes were obtained from ATCC. Cells were cultured using a standard protocol. Post-confluence cells were differentiated using a standard chemical cocktail mixture. Further cells were maintained in maturation medium and treated with native EGCG and Nano-EGCG at 10 µM for 48 hours. Cellular triglyceride levels were measured using an enzymatic method.

## Results

Nano-EGCG significantly (p<0.05) reduced triglyceride accumulation in mature adipocytes as compared to 1X PBS, native EGCG and its void counterpart. EGCG didn’t significantly reduce the cellular triglyceride accumulation as compared to 1XPBS.

## Conclusion

Data suggests that 10 uM of Nano-EGCG significantly reduced triglyceride accumulation as compared to 1X PBS, void counterpart and native EGCG.
